# A Single Session of tDCS Stimulation Can Modulate an EEG Microstate Associated With Anxiety in Patients With Depression

**DOI:** 10.1002/brb3.70580

**Published:** 2025-05-19

**Authors:** Keiichiro Nishida, Shota Minami, Tomonari Yamane, Satsuki Ueda, Banri Tsukuda, Shunichiro Ikeda, Daisuke Haruna, Masafumi Yoshimura, Tetsufumi Kanazawa, Thomas Koenig

**Affiliations:** ^1^ Department of Neuropsychiatry Osaka Medical and Pharmaceutical University Osaka Japan; ^2^ Department of Neuropsychiatry Kansai Medical University Osaka Japan; ^3^ Faculty of Clinical Psychology Kyoto Bunkyo University Kyoto Japan; ^4^ Department of Occupational Therapy, Faculty of Rehabilitation Kansai Medical University Osaka Japan; ^5^ Division of Systems Neuroscience of Psychopathology, Translational Research Center, University Hospital of Psychiatry University of Bern Bern Switzerland

## Abstract

**Purpose:**

Microstate analysis involves examining the temporal dynamics of electroencephalogram (EEG) signals and serves as a crucial method for exploring the neural basis of psychiatric disorders. This study investigates the effects of transcranial direct current stimulation (tDCS) on specific microstate parameter maps‐D and C in patients with depression, specifically targeting the dorsomedial prefrontal cortex (DMPFC) and left dorsolateral prefrontal cortex (DLPFC).

**Methods:**

We conducted an open‐label, between‐subject, crossover trial involving 19 patients clinically diagnosed with depression. A 1 mA electrical current was administered, with anodal stimulation specifically targeting the DMPFC or the left DLPFC. Microstate maps were derived from resting‐state EEG recordings obtained prior to and following the application of tDCS. The EEG data were categorized into five distinct microstate classes for subsequent analysis.

**Findings:**

The findings revealed a significant increase in the duration of microstate class D following stimulation in both groups, while microstate class C exhibited no notable changes. Additionally, a significant association was identified between the transition from microstate D to C and alterations in the State‐Trait Anxiety Inventory‐State (STAI‐S) scores after left DLPFC stimulation.

**Conclusion:**

Microstate map D appears to be associated with psychiatric disorders and executive functions, whereas map C may relate to the salience network and mind‐wandering. Our findings suggest that microstate maps D and C are responsive to tDCS stimuli, indicating their potential as objective tools for anxiety assessment. Employing transition‐focused parameters in EEG microstate analysis may enhance the tracking of rapidly fluctuating emotional states, rather than relying solely on duration metrics. Furthermore, the integration of non‐invasive brain stimulation techniques, such as tDCS, with EEG microstate analysis holds significant promise for elucidating the neural mechanisms involved in depression.

**Trial Registration:**

UMIN‐CTR Clinical Trial: UMIN000015046

## Graphical abstract

The findings demonstrated a significant increase in the duration of microstate class D following stimulation, while no changes were observed in class C. Moreover, transitions from microstate class C to D exhibited a correlation with the state‐trait anxiety inventory‐state anxiety (STAI‐SA) scores following dorsomedial prefrontal cortex (DMPFC) stimulation. In contrast, transitions from class D to C were linked to left dorsolateral prefrontal cortex (DLPFC) stimulation, albeit without a significant correlation. These results underscore the distinct and site‐specific effects of neural stimulation on anxiety‐related brain states.

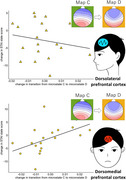

## 1 Introduction

Major depressive disorder (MDD) exerts a substantial societal burden and requires a comprehensive management approach. Biologically, depression is associated with dysfunction in brain regions involved in cognitive function, such as the prefrontal and motor cortices, and in regions involved in emotional processing, such as the amygdala and hippocampus. Furthermore, abnormalities in brain regions such as the dorsal cingulate gyrus and hypothalamus, which are associated with circadian rhythms, have also been observed, suggesting dysfunction at the whole‐brain level (Herrman et al. 2022).

Among the various approaches available to study brain function, one notable technique is electroencephalogram (EEG) microstate analysis, which was developed by Lehmann et al. (1987), (Christoph M Michel and Thomas Koenig, 2018). This method investigates the spatiotemporal dynamics of EEG signals to identify distinct, transiently stable global patterns of brain activity that are believed to reflect the activation of different neural networks. Microstate analysis has gathered attention as a valuable tool for exploring the neural mechanisms underlying psychiatric and neurological disorders (Das et al. 2022; Grieder et al. 2016; Koenig et al. 2002; Nishida et al. 2013; Tarailis et al. 2023).

Previous studies have reported alterations in microstate patterns in individuals with depression (Chivu et al. 2023). Research by Murphy et al. (2020) further revealed significant reductions in microstate D, a microstate map most closely associated with psychiatric disorders, in both individuals with MDD and those in remission when compared with healthy controls. Furthermore, a negative correlation was observed between microstate D duration and depression severity, as assessed using the Beck Depression Inventory.

Non‐invasive brain stimulation methods, such as electroconvulsive therapy (ECT), transcranial magnetic stimulation (TMS), and transcranial direct current stimulation (tDCS), provide targeted regulation of brain activity, and have shown efficacy in treating psychiatric and neurodegenerative disorders. Specifically, tDCS delivers a mild direct current through the scalp, thus modulating the excitability of underlying neuronal populations. Clinical trials of tDCS for depression continue to demonstrate its promising therapeutic potential despite some negative reports (Brunoni et al. 2017; Fregni et al. 2021).

Anodal stimulation of the left dorsolateral prefrontal cortex (DLPFC) is commonly used in tDCS studies in individuals with depression. However, in studies exploring emotional stimulation, such as repetitive TMS (rTMS), the medial prefrontal cortex (MPFC) has been targeted, suggesting the need for further investigation of optimal stimulation sites (Bakker et al. 2015; Colzato et al. 2015; Kreuzer et al. 2015). As a part of the emotional circuit, the MPFC plays a pivotal role in the integration of mental and physical states. Our previous research observed differences in stimulation effects based on the site of anodal tDCS; stimulation of the dorsomedial prefrontal cortex (DMPFC) resulted in reduced response times compared to stimulation of the left DLPFC in individuals with depression (Koshikawa et al. 2022).

Several studies have examined the relationship between transcranial electrical stimulation (tES) and microstates. For example, Kang et al. (2023) investigated individuals with low‐functioning autism and found differences in multiple microstate maps when comparing the pre‐ and post‐stimulation periods following tDCS. Another study involving healthy individuals utilized 40 Hz alternating current stimulation (tACS) and observed changes in microstate maps C and D (Gao et al. 2022). Regarding the relationship between depression and microstate maps, a recent meta‐analysis by Chivu et al. (2023) concluded that there is evidence of an association between microstate classes B and D and mood disorders. In addition, our previous study demonstrated that the anxiety‐reducing effect of tDCS on left DLPFC stimulation correlated with theta band activity in the rostral anterior cingulate cortex before stimulation. In another investigation, we found that the behavioral costs of tDCS of the DMPFC were lower than those of tDCS of the left DLPFC in patients with MDD. However, we did not examine neurophysiological mapping changes in the whole brain.

We hypothesized that the severity of depression is associated with microstate map D, the microstate most relevant to psychiatric disorders, and potentially with microstate map C because it was previously found to be affected by tES. Therefore, in this study, we examined these relationships and reported on the tDCS‐induced changes in microstate quantifiers and transitions, particularly in microstate maps D and C. Our investigation involved the analysis of pre‐post tDCS effects as a function of the stimulation site.

## 2 Methods

### 2.1 Study Design

The study protocol was approved by the Ethics Review Committee of the Kansai Medical University (UMIN000015046). All participants provided written informed consent, in accordance with the principles of the Declaration of Helsinki. The participants were recruited from September 2014 to April 2017. Other analyses have previously been published (Koshikawa et al. 2022; Nishida et al. 2019).

### 2.2 Procedure

**FIGURE 1:**
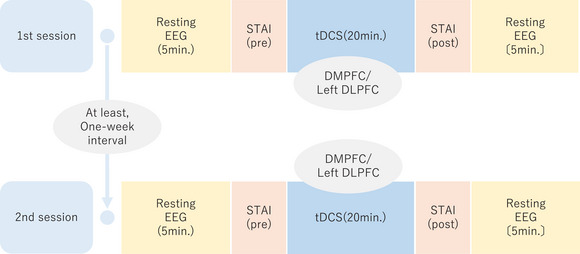
Timeline of the study. EEG: electroencephalography, STAI‐S: state‐trait anxiety inventory‐state anxiety, tDCS: transcranial direct current stimulation, DMPFC: dorsolateral medial prefrontal cortex, DLPFC: dorsolateral prefrontal cortex.

We employed between‐subjects, open‐label crossover trial design. The order of the stimulations was counterbalanced. Each participant was randomly assigned to receive either DMPFC or left DLPFC tDCS during the first session (Figure 1). The participants underwent tDCS at another site during the second session. There was an interval of at least one week between tDCS sessions.

Twenty participants diagnosed with MDD, as defined in the Diagnostic and Statistical Manual of Mental Disorders, Fourth Edition (DSM‐IV‐TR) (American Psychiatric Association 2000), were included (Table 1). The severity of illness was assessed using the Hamilton Depression Rating Scale 17 (HAM‐D 17) and MADRS (Montgomery Asberg Depression Rating Scale). One patient was excluded because his EEG data were incomplete owing to imperfect recording conditions. All the participants were right‐handed and held at least one high school diploma or an equivalent advanced degree.

**TABLE 1:** Demographic data of the study participants.

Session	DMPFC	Left DLPFC
Sample size	19	
Sex: male/female	12/7	
Drug treatment		
No	1	
One antideoressant	8	
Two antidepressant	4	
Three antidepreaant	4	
Four antidepressant	2	
Benzodiazepine	14	
Mood stabilizer	3	
Age‐ years:mean ± SD	56.05 ± 13.39	
Education period	14.53 ± 2.11	
Number of previous episodes: mean ± SD	2.26 ± 0.91	
HAM‐D17 score on the day of the session: mean ± SD	14.05 ± 4.92	13.90 ± 4.89
MADRS score on the day of the session: mean ± SD	23.26 ± 6.64	22.90 ± 6.16
STAI‐S score on the day of the session: mean ± SD		
Pre test	46.32 ± 8.57	48.70 ± 9.13
Post test	44.95 ± 9.86	46.25 ± 9.54
STAI‐T score on the day of the session: mean ± SD		
Pre test	56.84 ± 12.65	55.65 ± 10.72
Post test	55.74 ± 11.97	54.60 ± 12.35

Experienced psychiatrists conducted a comprehensive diagnostic process, using the Structured Clinical Interview for DSM‐IV‐TR (First et al., 2002), and physical examinations, and endocrinal blood tests to rule out neurocognitive disorder and endocrine‐related mental disorders. The exclusion criteria encompassed patients with a history of dementia, schizophrenia, substance dependence, epilepsy, or head trauma, attachment disorders, and post‐traumatic stress disorder (PTSD) were also excluded. Furthermore, to ensure that depression was the primary diagnosis and not eventually the consequence of another disorder, individuals with comorbid anxiety disorders, such as generalized anxiety disorder, panic disorder, and phobias, were excluded through dimensional approach.

### 2.3 EEG Recording

Resting and eyes‐closed EEG were recorded using the EEG‐1200 Nihon Kohden (Tokyo, Japan) system. A 64 ch Ag/AgCl sintered Waveguard Original EEG cap from ANT Neuro (the Netherlands) was used for the recordings. Data were sampled at 500 Hz and recorded with a 0.5 Hz low‐pass filter and a 60 Hz high‐pass filter. The duration of each recording was 5 min. We recorded EEG twice during one session: before and immediately after the tDCS period (Figure 1).

### 2.4 EEG Preprocessing

We organized the 5 min EEG recordings into two minutes each using an algorithm to extract epochs with few artifacts within the LORETA software (https://www.uzh.ch/keyinst/). In the LORETA program, the mean amplitude, global field power, skewness, and kurtosis over the entire recording period was calculated, and periods exceeding 5 standard deviations above the mean were identified as artifacts objectively. These periods were also carefully inspected visually and excluded. Among the remaining periods, the earliest 2 min of the available time slot were used for analysis to avoid confounding effects of drowsiness that may have occurred later in the recording. EEGs were reconstructed using independent component analysis (ICA) with eye movement rejection to remove components corresponding to eye movement artifacts. For this task, the ICA Independent Component Analysis within the Brain Vision Analyzer 2 (Brain Products GmbH, Gilching, Germany) was used.

### 2.5 EEG Microstate Analysis

Microstate analysis was performed using MATLAB for EEGLAB (Version 2021R) with a plug‐in developed by Thomas Koenig (http://www.thomaskoenig.ch/index.php/work/software). For this analysis, the data was downsampled to 30 channels. This downsampling was done because we wanted to ensure compatibility with other data that contains only 30 channels, and because a preliminary microstate analysis with 64 channels yielded implausible results. We ensured the smoothness of the maps through visual inspection by two experienced staff members using 30 channels (Fp1, Fp2, F7, T3, Fz, F4, F8, FT7, FC3, FCz, FC4, FT8, T3, C3, Cz, C4, T4, TP7, CP3, CPz, CP4, TP8, T5, P3, Pz, P4, T6, O1, O2, and O2).

As we had cleared the hypothesis regarding microstate classes C and D, we aimed to identify microstate clusters that contained these two maps. K‐means clustering was used to construct sets of four to seven microstate maps for each participant based on data filtered at 2–20 Hz. Next, we combined the microstate maps across the subjects into a grand mean, permuting individual maps such that they shared the maximal amount of variance across the subjects. The topographies of the five microstate class solutions were the first to show to identify microstate classes C and D, and were therefore used for the rest of the analysis. We then backfitted these grand‐mean template maps to the GFP peaks of the individual EEGs, interpolated the microstate assignment between these peaks based on the nearest neighbor, and extracted the basic microstate parameters (duration, contribution, and occurrence). We further quantified the transition from map D to map C and from map C to map D, correcting for the transition probabilities expected from microstate occurrences alone (Lehmann et al. 2005).

### 2.6 tDCS

The tDCS was delivered using a battery‐operated stimulator (DC Stimulator Plus; NeuroConn, Ilmenau, Germany). We applied a 1 mA electrical current through conductive rubber electrodes (2 cm^2, circular) secured with an adhesive EEG‐conductive paste. Anodal stimulation targeted the left DLPFC (F5, 10–10 EEG international electrode placement) or DMPFC (AFz, 10‐10 EEG international electrode placement). The cathode (5 × 7 cm^2) was positioned on the left shoulder. A direct current was delivered for 20 min in the resting state.

### 2.7 Psychological Test

The state‐trait anxiety inventory (STAI) is a self‐administered assessment tool to evaluate the severity of anxiety (Spielberger et al. 1971). It differentiates between state anxiety, a transient emotional condition triggered by specific stressors, and trait anxiety, a stable and enduring predisposition to experience anxiety. Each subscale consists of 20 items that address somatic, affective, and cognitive dimensions of anxiety. The total score for each subscale ranges from 20 to 80, with higher scores indicating greater levels of anxiety. In this study, we used the STAI ‐state (STAI‐S) to assess temporary anxiety levels before and after tDCS. The severity of depression was assessed using the Montgomery‐Åsberg depression rating scale (MADRS) (Montgomery and Åsberg 1979). The MADRS total score ranges from 0 to 60, with the following cutoff points commonly applied: 0–6 indicating no depression.

### 2.8 Statistical Analysis

All pre‐post comparisons were statistically assessed using the Wilcoxon signed‐rank exact test, as the Shapiro‐Wilk test indicated a non‐normal distribution. P values were corrected using the Benjamini–Hochberg procedure (BH). Based on the arguments made in the introduction, we were particularly interested in stimulation‐site‐specific changes in the duration and contribution of microstate classes D and C, and changes in the transitions between classes C and D. Correlations between these changes and changes in the STAI were tested using Pearson's product‐moment correlation, as the Shapiro‐Wilk test indicated a normal distribution. Finally, to contribute to the ongoing research on baseline alterations of EEG microstates in depression, we correlated the total pre‐stimulation MADRS score at the first visit with the occurrence and duration of microstate classes A and D using Pearson's product‐moment correlation. As a supplementary analysis, we compared the duration and contribution of map E before and after stimulation using the Wilcoxon signed‐rank exact test to examine the characteristics of class E. Furthermore, we investigated the Pearson's product‐moment correlation between the changes in transitions between class C and class E, and the changes in STAI‐S, following the approach of the previous analysis.

All analyses were performed on software R 4.4.1 (R Core Team 2024), lme4 (Bates et al. 2015), and lmerTset (Kuznetsova et al. 2017).

## 3 Results

### 3.1 Characteristics of the Participants, Grand‐Mean Microstate Maps, and Mean Microstate Parameters

**FIGURE 2:**
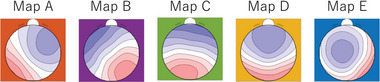
The five grand mean microstate maps obtained in this study.

Table 1 shows the characteristics of the study participants, while Figure 2 presents the grand‐mean microstate maps. These maps reproduced previously obtained microstate maps very well (Koenig et al. 2024; Tarailis et al. 2024). Table 2 shows the parameters of the microstate maps A, B, C, D, and E. The obtained microstate durations were within the expected range, and a preponderance of microstate class C was observed, which is typical of resting‐state EEG in adults. Finally, Table 3 shows the deviations of the transitions from maps C to D and from maps D to C after correcting for the proportion of transitions expected from the occurrences of different microstate classes.

**TABLE 2:** The map parameters (duration, contribution, and occurrence) in DMPFC and left DLPFC. DMPFC: Dorsolateral medial prefrontal cortex, DLPFC: Dorsolateral prefrontal cortex.

Parameters	Session	Timing	Map A		Map B		Map C		Map D		Map E	
			Mean	SD	Mean	SD	Mean	SD	Mean	SD	Mean	SD
Duration (ms)	DMPFC	Pre stimulation	57.97	6.06	55.68	6.65	58.28	8.83	72.90	22.88	63.76	11.60
		Post stimulation	58.31	6.64	53.37	8.33	58.05	8.62	79.11	27.52	64.36	15.14
	Left DLPFC	Pre stimulation	57.85	7.43	57.00	9.11	60.39	10.80	72.47	29.91	62.59	11.23
		Post stimulation	56.85	7.67	54.79	7.98	60.38	13.44	83.56	42.56	63.28	10.94
Contribution (ms/s)	DMPFC	Pre stimulation	0.18	0.05	0.16	0.05	0.18	0.05	0.27	0.10	0.21	0.06
		Post stimulation	0.18	0.06	0.14	0.05	0.17	0.04	0.30	0.10	0.21	0.08
	Left DLPFC	Pre stimulation	0.18	0.05	0.16	0.06	0.20	0.08	0.25	0.11	0.21	0.06
		Post stimulation	0.16	0.05	0.14	0.06	0.19	0.09	0.30	0.12	0.20	0.07
Occurrence/s	DMPFC	Pre stimulation	3.19	0.95	2.94	0.94	3.17	0.77	3.65	0.53	3.28	0.71
		Post stimulation	3.11	1.00	2.71	0.92	2.94	0.77	3.91	0.47	3.28	0.78
	Left DLPFC	Pre stimulation	3.05	0.95	2.85	0.95	3.34	0.98	3.49	0.81	3.42	0.89
		Post stimulation	2.87	0.92	2.59	0.99	3.17	0.95	3.89	0.74	3.24	1.02

**TABLE 3:** Transition of the map from C to D, and from D to C in DMPFC and left DLPFC. DMPFC: Dorsolateral medial prefrontal cortex, DLPFC: Dorsolateral prefrontal cortex.

Session	DMPFC				Left DLPFC			
	Pre stimulation		Post stimulation		Pre stimulation		Post stimulation	
	Mean	SD	Mean	SD	Mean	SD	Mean	SD
Map from C to D	0.00514	0.01201	0.00317	0.00961	0.00435	0.00989	0.00270	0.01149
Map from D to C	0.00424	0.00886	0.00279	0.00948	0.00350	0.00894	0.00487	0.01203

### 3.2 Stimulation Effects

**FIGURE 3:**
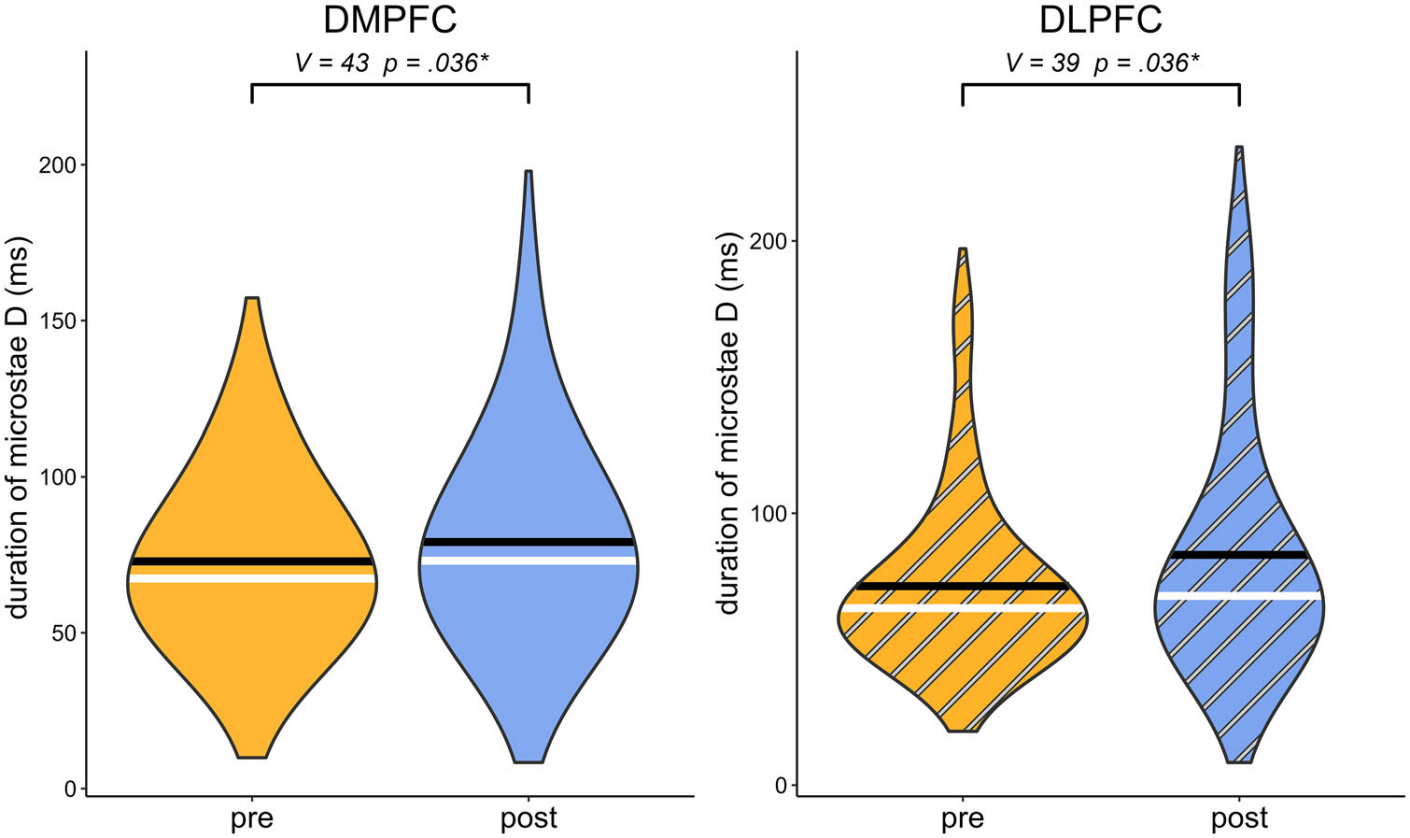
Violin plots of the duration of microstate class D on DMPFC stimulation (left) and left DLPFC stimulation (right). DMPFC: dorsolateral medial prefrontal cortex, DLPFC: dorsolateral prefrontal cortex.

**FIGURE 4:**
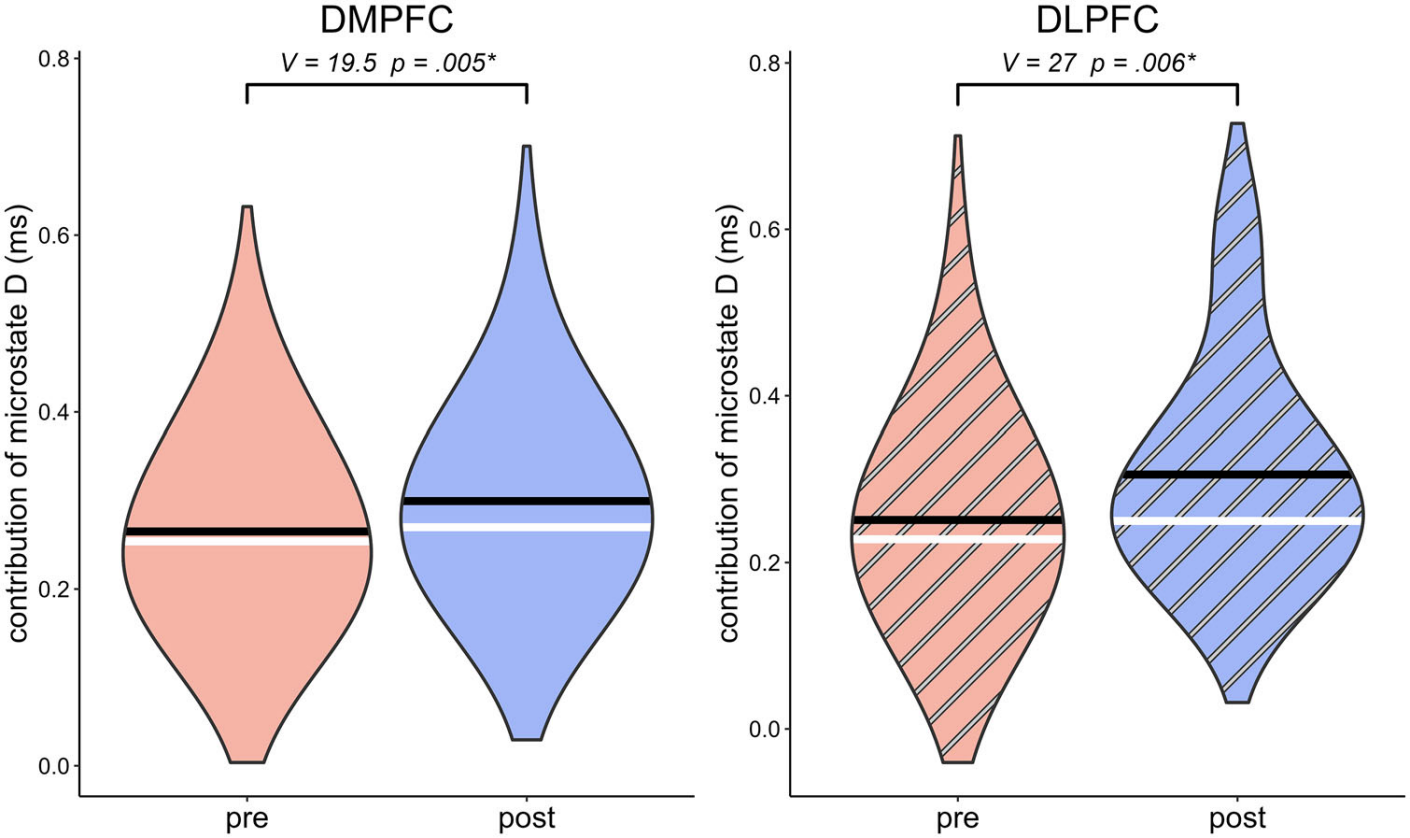
The violin plots of the contribution of microstate class D on DMPFC stimulation (left) and left DLPFC stimulation (right). DMPFC: dorsolateral medial prefrontal cortex, DLPFC: dorsolateral prefrontal cortex.

Table 4 shows the outcome of the pre‐post comparisons of microstate class D and C parameters as a function of the stimulation site. There was a significant difference in both the DMPFC (V = 43, p = 0.036) and left DLPFC stimulation (V = 39, p = 0.036) in the comparison of the duration of class D microstates before and after the intervention. The violin plots of these duration effects are shown in Figure 3. There was also a significant difference in DMPFC (V = 19.5, p = 0.005) and in DLPFC stimulation (V = 27, p = 0.006) in the MDD group when comparing the contribution of class D before and after the intervention. The violin plots of these contribution effects are shown in Figure 4. There were no significant effects associated with microstate class C for any combination of microstate parameters and stimulation sites (all p > 0.100). The results of the statistical analysis of the transitions between microstate classes C and D are listed in Table 5. However, none of these effects reached statistical significance.

**TABLE 4:** Comparison of duration and contribution of maps D, before and after stimulation.

Parameters	Stimulation	Statistic(*V*)	*p* value
Duration of microstate map D	DMPFC	43	0.036
	Left‐DLPFC	39	0.036
Contribution of microstate map D	DMPFC	19.5	0.005
	Left‐DLPFC	27	0.006
Duration of microstate map C	DMPFC	97	0.953
	Left‐DLPFC	102.5	0.763
Contribution of microstate map C	DMPFC	135.5	0.103
	Left‐DLPFC	112.5	0.481

**TABLE 5:** Comparison of changes in the microstate transition before and after stimulation.

Transition	Stimulation	Statistic(*V*)	*p* value
From microstate map C to microstate map D	DMPFC	118	0.374
	Left‐DLPFC	118	0.374
From microstate map D to microstate map C	DMPFC	108	0.623
	Left‐DLPFC	77	0.490

### 3.3 Correlations Between Map Changes and STAI‐S Changes

Correlations between changes in STAI‐S scores and changes in duration and contribution following tDCS intervention are shown in Table 6. None of these correlations were statistically significant.

**TABLE 6:** Correlation stimulation induced changes in microstate parameters and state‐trait anxiety inventory‐state anxiety (STAI‐S) score changes.

Parameters	Stimulation	Statistic(*t*)	*df*	*r*	*p* value
Duration of microstate map D	DMPFC	0.155	17	0.038	0.878
	Left‐DLPFC	0.624	17	####	0.541
Contribution of microstate map D	DMPFC	0.135	17	0.033	0.894
	Left‐DLPFC	−0.83	17	####	0.412
Duration of microstate map C	DMPFC	−0.199	17	####	0.844
	Left‐DLPFC	−0.475	17	####	0.640
Contribution of microstate map C	DMPFC	0.62	17	0.149	0.544
	Left‐DLPFC	−0.228	17	####	0.822

**FIGURE 5:**
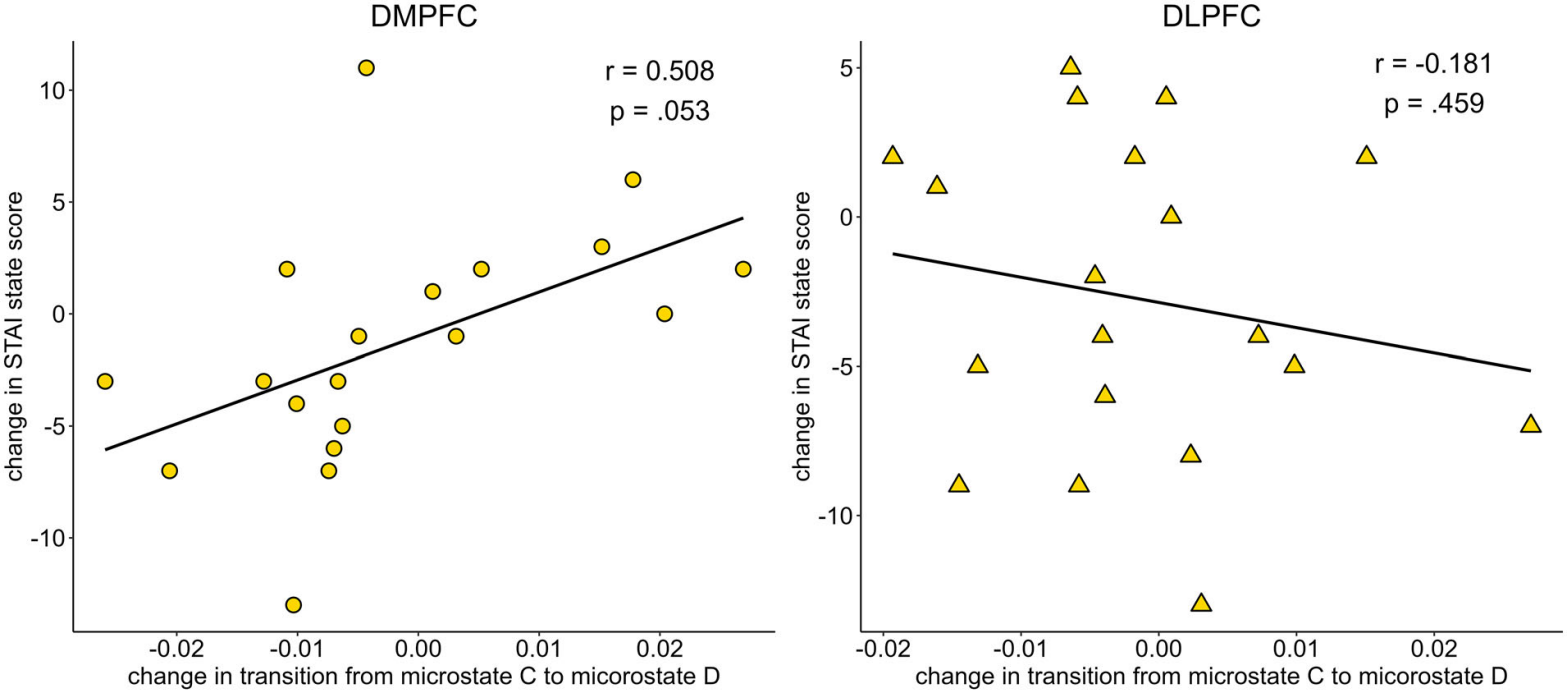
Scatterplot of changes in the state‐trait anxiety inventory‐state anxiety (STAI‐S) score and change in transition from microstate map from map C to map D after DMPFC stimulation (left) and left DLPFC stimulation(right). DMPFC: dorsolateral medial prefrontal cortex, DLPFC: dorsolateral prefrontal cortex.

**FIGURE 6:**
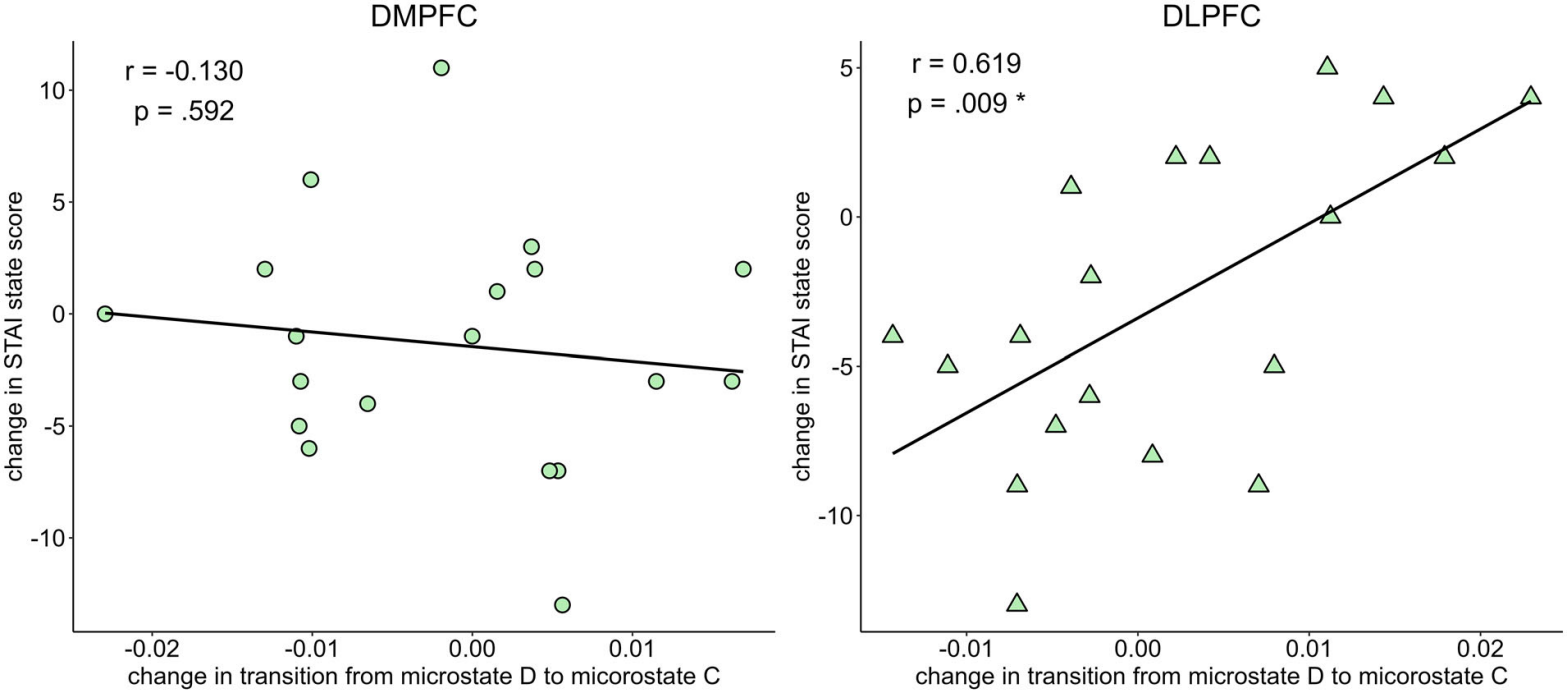
Scatterplot of changes in the state‐trait anxiety inventory‐state anxiety (STAI‐S) score, and the change in transition from microstate map from D to map C, after DMPFC stimulation (left) and after left DLPFC stimulation (right). DMPFC: dorsolateral medial prefrontal cortex, DLPFC: dorsolateral prefrontal cortex.

The results of the analysis correlating stimulation‐induced changes in microstate transitions with changes in STAI‐S scores are shown in Table 7. Changes in STAI‐S correlated marginally with changes in the transition from C to D after DMPFC stimulation (r = 0.508, p = 0.053), and significantly with changes in the transition from D to C after left DLPFC stimulation (r = .619, p = 0.009) (Figures 5 and 6).

**TABLE 7:** Correlation between tDCS pre‐ and post‐intervention changes in state‐trait anxiety inventory‐state anxiety (STAI‐S) score and transitions.

Transition	Stimulation	Statistic(*t*)	*df*	*r*	*p* value
From microstate map C to microstate map D	DMPFC	2.429	17	0.508	0.053
	Left‐DLPFC	−0.757	17	−0.181	0.459
From microstate map D to microstate map C	DMPFC	−0.541	28	−0.130	0.595
	Left‐DLPFC	3.253	17	0.619	0.009

### 3.4 Correlations Between Pre‐Stimulation EEG Parameters the MADRS Score

We found a significant correlation between the MADRS score at pre‐tDCS and the occurrence of microstate map A (t = 2.22, df = 17, r = 0.473, p = 0.041), but no significant correlation between the duration of microstate class D and MADRS (t = ‐2.019, df = 17, r = ‐0.440, p = 0.060).

### 3.5 Characteristics of the Class E

There were no significant differences in the duration or contribution of microstate class E between the pre‐ and post‐stimulation conditions for both DMPFC (duration: V = 92, p = 0.921, contribution: V = 82, p = 0.623) and left DLPFC (duration: V = 89, p = 0.829, contribution: V = 96, p = 0.647) stimulation (Supplementary Table 1, Supplementary Figure 1, and Supplementary Figure 2). Pearson's product‐moment correlation analysis was conducted to examine the relationship between changes in transitions between class C and class E and changes in STAI‐S; however, no significant correlations were observed overall (Supplementary Figure 3 and Supplementary Figure 4). Correlation analysis showed that changes in STAI‐S were not significantly associated with changes in the transition from C to E after DMPFC stimulation (r = .522, p = 0.088) or left DLPFC stimulation (r = .160, p = 0.684). Similarly, no significant correlations were found between changes in STAI‐S and changes in the transition from E to C after DMPFC stimulation (r = .319, p = 0.365) or left DLPFC stimulation (r = .015, p = 0.952).

## 4 Discussion

The results of the present study confirm earlier reports that direct brain stimulation can systematically alter brain EEG resting microstates. Our study further extends previous findings to the application of tDCS to two regions relevant for the treatment of depression and shows that this stimulation resulted in significant prolongation of the duration and contribution of microstate class D, a brain state often implicated in psychiatric conditions. However, there was no significant change in duration C and Contribution C in either treatment group. In addition, the change in transition from C to D significantly correlated with the change in STAI‐S during DMPFC stimulation, and the change in transition from D to C significantly correlated with the change in STAI‐S after left DLPFC stimulation.

Microstate class D is the microstate class that has been most closely related to mental illness. In particular, the duration of map D is the most important factor related to executive processing when using microstate analyses (Koenig et al. 2024; C. M. Michel and T. Koenig 2018). Murphy et al. (2020) further found that the presence of microstate D was reduced in depression, the mean duration and contribution correlated with severity, and that the duration of microstate D was longer after treatment than before treatment. Another study has further shown that the duration and coverage of microstate class D increased after SSRI treatment in depressed patients (Lei et al. 2022). The fact that the duration of map D in our study was longer after DMPFC and left DLPFC stimulation is consistent with these previous studies.

The role of microstate D alterations in the context of depression treatment is further supported by systematic changes in the microstates elicited by tDCS. In the past, we pointed out that transitions from map C to map D and from map D to map C may reflect disease symptoms (Nishida et al. 2013). Interestingly, in the present study, we found a strong correlation between changes in the STAI‐S and changes in transitions between maps C and D. One study reported that microstate map C is associated with mind‐wandering and self‐related thoughts, while microstate map D is associated with internally oriented processing, in addition to the executive network (Bréchet et al. 2021). Although hypothetical, it is possible that the interactions between microstate maps C and D are related to rumination, which has been the focus of considerable attention in depression. Although speculative, it has recently been suggested that the ventral MPFC is associated with autonomic effects through the hypothalamus (Kim et al. 2018) and somatic processes (Davey and Harrison 2022). In our study, using the same dataset as the current study, DMPFC stimulation with tDCS in patients with depression improved conflict resolution, whereas task switching was not affected (Koshikawa et al. 2022). Interestingly, Pan et al. (2020) further reported a decrease in the duration of microstate map C and coverage of microstate map C, along with a decrease in rumination, which is presumed to be related to internal conflicts. The fact that transitions from map C to D in DMPFC stimulation and transitions from map D to C in left DLPFC stimulation correlated with the amount of change in anxiety may suggest a relationship between these ruminations.

Finally, we discuss the microstate class A. Damborská et al. (2019) showed that the occurrence of map A was significantly correlated with MADRS scores. Our study also found a correlation between the occurrences of microstate map A and the severity of depression. Kikuchi et al. (2011) reported that the duration of microstate map A was prolonged in patients with panic, in addition to a decrease in microstate occurrence. Atluri et al. (2018) also mentioned the use of microstate map A in electrocompulsive therapy for patients with depression. As such, the role of microstate A in depressed mood and anxiety needs to be examined in future studies.

## 5 Limitation

This study had three limitations. Although the number of participants was determined based on our previous study, the sample size may limit the generalizability of the findings. Further research with larger cohorts is warranted, and the lack of a double‐blind protocol and the absence of a sham control may lead to experimenter or participant bias. Finally, the recruitment of mainly mild cases, which narrows its applicability to severe scenarios. Further studies are required to address these limitations.

## 6 Conclusion

Our findings revealed that microstate maps D and C were responsive to tDCS stimuli, potentially serving as crucial objective tools for anxiety assessment. EEG microstate analysis may warrant the application of transition‐focused parameters, as opposed to duration or other metrics, for the accurate tracking of rapidly fluctuating events such as emotions. Moreover, the integration of non‐invasive brain stimulation techniques, such as tDCS, with EEG microstate analysis presents considerable promise for revealing the neural mechanisms implicated in depression.

## Author Contributions

**Keiichiro Nishida**: conceptualization, data curation, formal analysis, resources, supervision, validation, writing–original draft, writing–review and editing. **Shota Minami**: data curation, formal analysis, funding acquisition. **Tomonari Yamane**: formal analysis, methodology. **Satsuki Ueda**: formal analysis, methodology. **Banri Tsukuda**: data curation, methodology, resources. **Shunichiro Ikeda**: software, supervision. **Daisuke Haruna**: data curation, formal analysis, software. **Masafumi Yoshimura**: supervision, validation. **Tetsufumi Kanazawa**: methodology, supervision. **Thomas Koenig**: investigation, software, supervision, validation.

## Conflicts of Interest

Keiichiro Nishida belongs to the committee on Psychiatric Medical Devices and Brain Stimulation Committee, Japanese Society of Clinical Neurophysiology. The other authors declare that they have no competing interests.

### Peer Review

The peer review history for this article is available at https://publons.com/publon/10.1002/brb3.70580.

## Supporting information



Supplementary Table 1 Comparison of duration and contribution of maps E, before and after stimulation
Supplementary Figure 1 Violin plots of the duration of microstate class E on DMPFC stimulation (left) and left DLPFC stimulation (right).
Supplementary Figure 2 The violin plots of the contribution of microstate class E on DMPFC stimulation (left) and left DLPFC stimulation (right).
Supplementary Figure 3 Scatterplot of changes in the state‐trait anxiety inventory‐state anxiety (STAI‐S) score and change in transition from microstate map from map C to map E after DMPFC stimulation (left) and left DLPFC stimulation(right).
Supplementary Figure 4 Scatterplot of changes in the state‐trait anxiety inventory‐state anxiety (STAI‐S) score, and the change in transition from microstate map from E to map C, after DMPFC stimulation (left) and after left DLPFC stimulation (right).

## Data Availability

The data that support the findings of this study are available from the corresponding author upon reasonable request. The data are not publicly available due to privacy or ethical restrictions.
