# Supplementary material for: A Single Session of tDCS Stimulation Can Modulate an EEG Microstate Associated With Anxiety in Patients With Depression
Source: Brain Behav. 2025 May 19;15(5):e70580. doi: 10.1002/brb3.70580 (PMC12086299; doi:10.1002/brb3.70580)

**A single session of tDCS stimulation can modulate an EEG microstate associated with anxiety in patients with depression**

**Supplementary information**

Keiichiro Nishida, Shota Minami, Tomonari Yamane, Satsuki Ueda, Banri Tsukuda, Shunichiro Ikeda, Daisuke Haruna, Masafumi Yoshimura, Tetsufumi Kanazawa, and Thomas Koenig

**Supplementary Table 1.: Comparison of Duration and Contribution of Maps E, before and after stimulation**

**Supplementary Figure 1.: Violin plots of the duration of microstate class E on DMPFC stimulation (left) and left DLPFC stimulation (right). DMPFC: dorsolateral medial prefrontal cortex, DLPFC: dorsolateral prefrontal cortex**


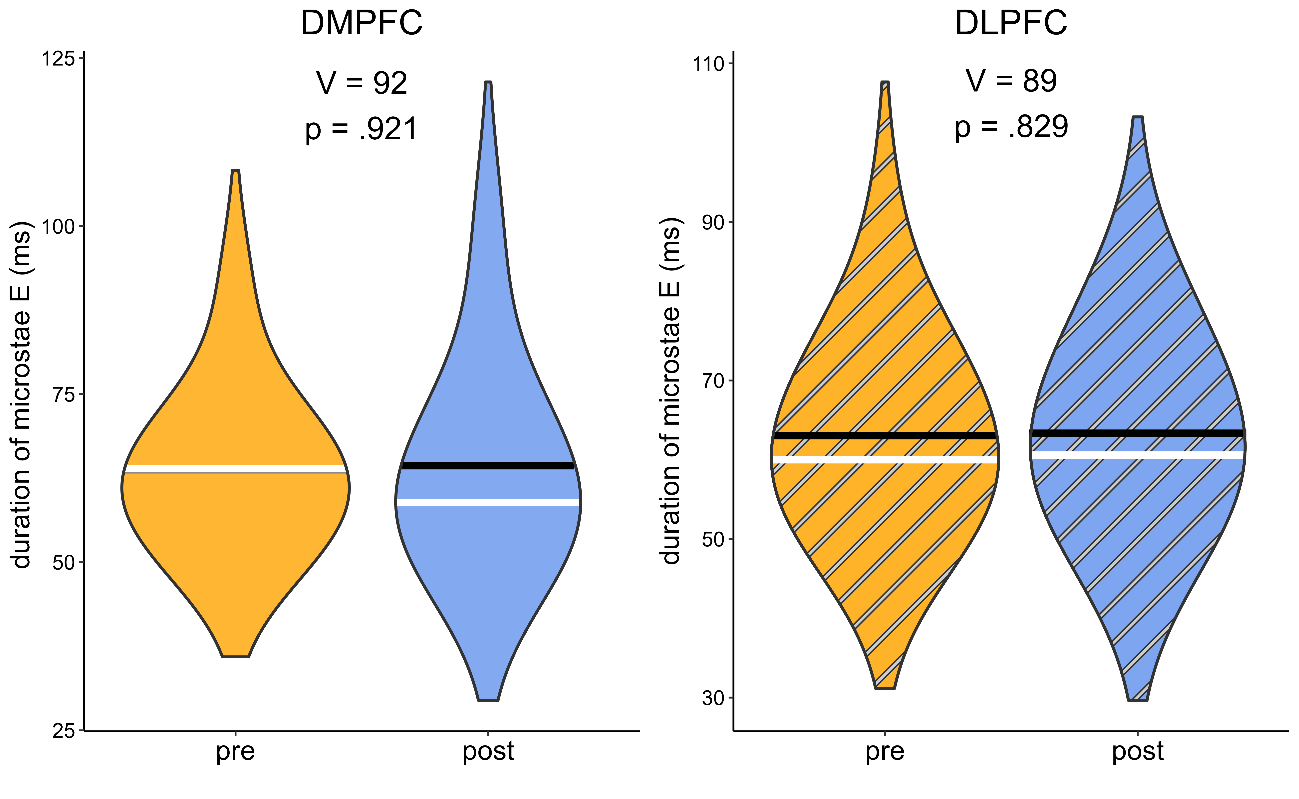


**Supplementary Figure 2.: The violin plots of the contribution of microstate class E on DMPFC stimulation (left) and left DLPFC stimulation (right). DMPFC: dorsolateral medial prefrontal cortex, DLPFC: dorsolateral prefrontal cortex**


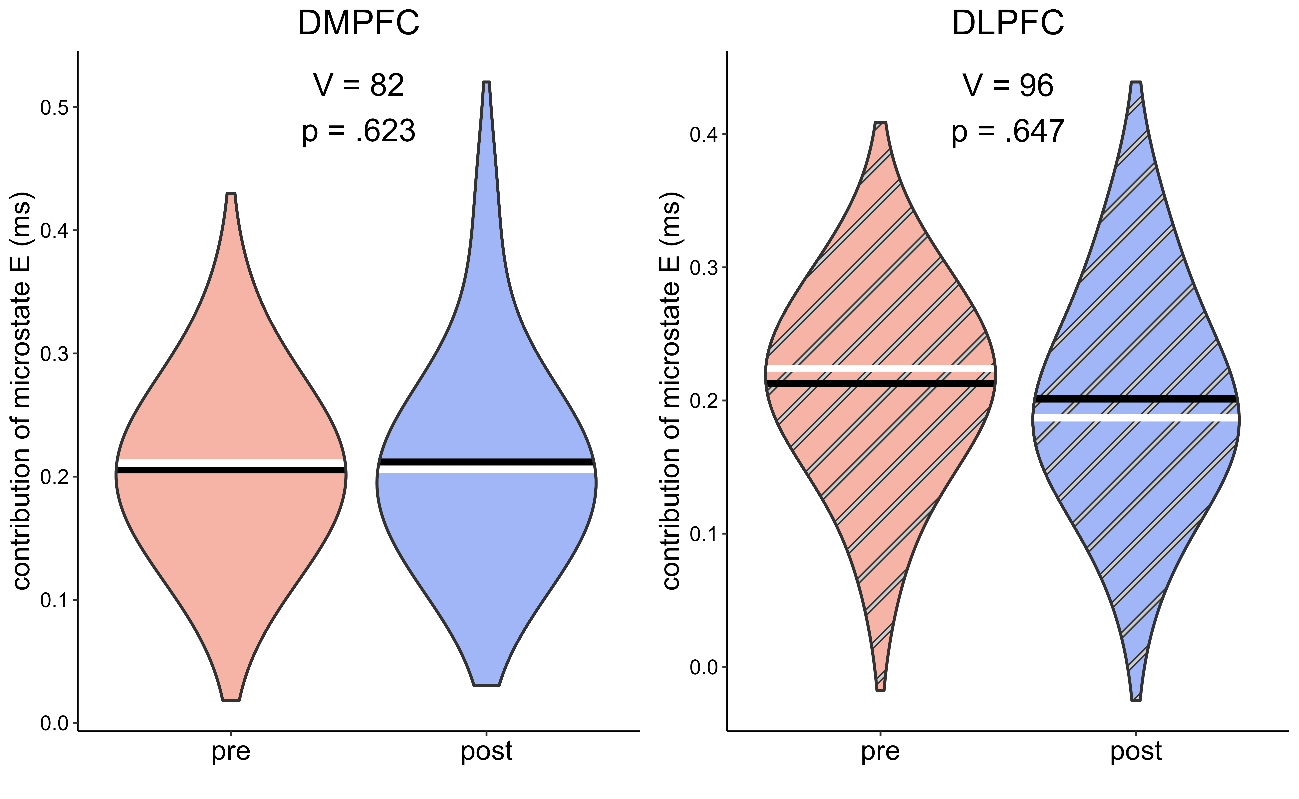


**Supplementary Figure 3.: Scatterplot of changes in the State-Trait Anxiety Inventory-State Anxiety (STAI-S) score and change in transition from microstate map from map C to map E after DMPFC stimulation (left) and left DLPFC stimulation(right). DMPFC: dorsolateral medial prefrontal cortex, DLPFC: dorsolateral prefrontal cortex**


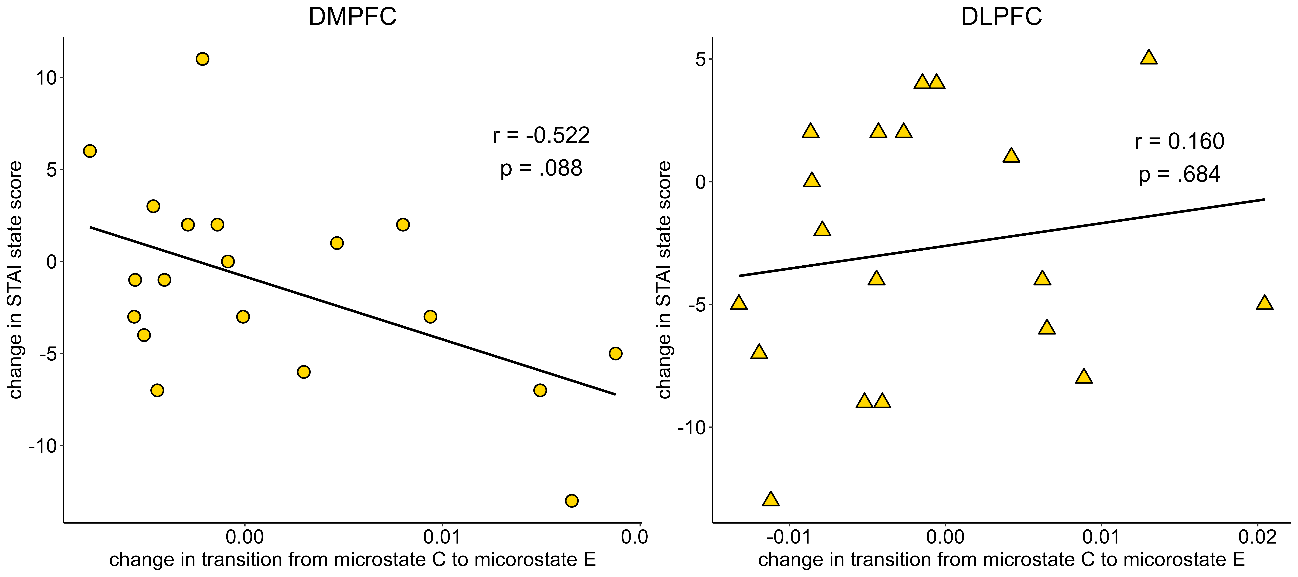


**Supplementary Figure 4.:** **Scatterplot of changes in the State-Trait Anxiety Inventory-State Anxiety (STAI-S) score, and the change in transition from microstate map from E to map C, after DMPFC stimulation (left) and after left DLPFC stimulation (right). DMPFC: dorsolateral medial prefrontal cortex, DLPFC: dorsolateral prefrontal cortex**


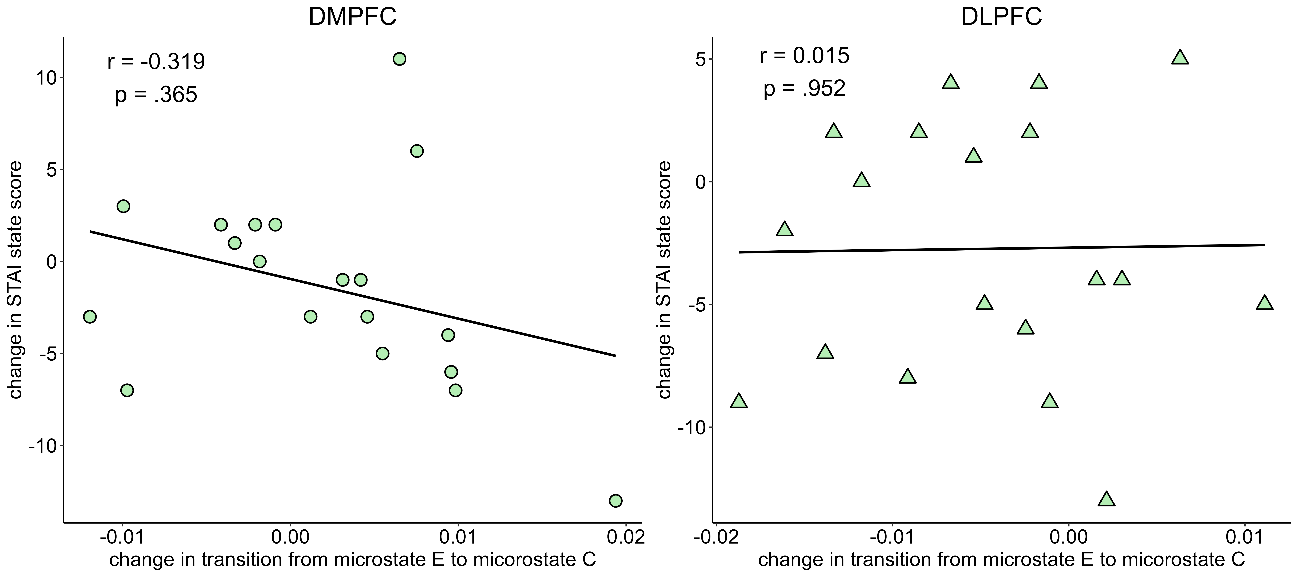

Supplement: Supplementary file 1 — Supplementary Table 1 Comparison of duration and contribution of maps E, before and after stimulation Supplementary Figure 1 Violin plots of the duration of microstate class E on DMPFC stimulation (left) and left DLPFC stimulation (right). Supplementary Figure 2 The violin plots of the contribution of microstate class E on DMPFC stimulation (left) and left DLPFC stimulation (right). Supplementary Figure 3 Scatterplot of changes in the state‐trait anxiety inventory‐state anxiety (STAI‐S) score and change in transition from microstate map from map C to map E after DMPFC stimulation (left) and left DLPFC stimulation(right). Supplementary Figure 4 Scatterplot of changes in the state‐trait anxiety inventory‐state anxiety (STAI‐S) score, and the change in transition from microstate map from E to map C, after DMPFC stimulation (left) and after left DLPFC stimulation (right). [file BRB3-15-e70580-s001.docx]
